# Patient-Physician Communication in the Era of Mobile Phones and Social Media Apps: Cross-Sectional Observational Study on Lebanese Physicians’ Perceptions and Attitudes

**DOI:** 10.2196/medinform.8895

**Published:** 2018-04-06

**Authors:** Fady Daniel, Suha Jabak, Roula Sasso, Yara Chamoun, Hani Tamim

**Affiliations:** ^1^ Department of Internal Medicine American University of Beirut Medical Center American University of Beirut Beirut Lebanon; ^2^ Department of Emergency Medicine American University of Beirut Medical Center American University of Beirut Beirut Lebanon; ^3^ Hotel-Dieu de France Medical Center Department of Psychiatry Université Saint Joseph Beirut Lebanon; ^4^ Biostatistics Unit, Clinical Research Institute American University of Beirut Medical Center American University of Beirut Beirut Lebanon

**Keywords:** social media, communication, patient-physician communication, technology use

## Abstract

**Background:**

The increased prevalence of virtual communication technology, particularly social media, has shifted the physician-patient relationship away from the well-established face-to-face interaction. The views and habits of physicians in Lebanon toward the use of online apps and social media as forms of patient communication have not been previously described.

**Objective:**

The aim of this study is to describe the views of Lebanese physicians toward the use of social media and other online apps as means of patient communication.

**Methods:**

This was a cross-sectional observational study using an online survey that addressed physicians’ perceptions on the use of virtual communication in their clinical practice. The study took place between April and June 2016, and was directed toward physicians at the American University of Beirut Medical Center.

**Results:**

A total of 834 doctors received the online survey, with 238 physicians completing the survey. Most of the participants were from medical specialties. Most responders were attending physicians. Less than half of the respondents believed that Web-based apps and social media could be a useful tool for communicating with patients. Email was the most common form of professional online app, followed by WhatsApp (an instant messaging service). The majority of participants felt that this mode of communication can result in medicolegal issues and that it was a breach of privacy. Participants strictly against the use of virtual forms of communication made up 47.5% (113/238) of the study sample.

**Conclusions:**

The majority of physicians at the American University of Beirut Medical Center are reluctant to use virtual communication technology as a form of patient communication. Appropriate policy making and strategies can allow both physicians and patients to communicate virtually in a more secure setting without fear of breaching privacy and confidentiality.

## Introduction

The medical world is changing and the use of online communication is becoming more abundant [1]. The widespread availability of Internet-connected mobile phones has introduced new virtual ways of communication between individuals including social media apps. Patients are now emailing, text messaging, chatting, and video chatting with their physicians to speed up their health care [2]. The patient-physician relationship that has been traditionally implemented by face-to-face communication may be slowly shifting toward a more virtual form of communication [3,4] because of the increased use of social media and social networking.

Online physician-patient communication can improve health care by enhancing patient education [5], improving patient compliance to medication use, bettering adherence to physician recommendations [6,7], and facilitating easier patient follow-up for chronic diseases [8].

Currently, there are no studies in Lebanon that describe physicians’ perceptions on the use of virtual communication in patient care. Since social media is becoming a more abundant form of communication in clinical practice, we designed this study with the aim of describing how social media and networking among other virtual communication technologies are regarded and utilized by practicing and training physicians in Lebanon.

## Methods

### Participants

Our study was a cross-sectional observational study that took place between April 2016 and June 2016 at the American University of Beirut Medical Center (AUBMC), a large tertiary care academic medical center in Beirut.

Practicing and training physicians from all medical specialities at AUBMC with an email address in the AUBMC directory were eligible to participate in our study. Online questionnaires were distributed via an online survey tool (Lime survey).

Responders to the questionnaires remained anonymous and individuals who completed the online questionnaire did not receive any financial compensation. The institutional review board committee at AUBMC approved the study.

### Questionnaire

Our research team developed the questionnaire after a thorough literature review and the questions were developed based on the currently available literature and our basic research question. The questionnaire included 22 questions that examined general demographics as well as three scopes of the doctor-patient online interaction: (1) extent of participants’ personal use of online apps and social media, such as email, LinkedIn (a business community networking site), Instagram (a photo-sharing Web service), WhatsApp (an instant messaging service), and Facebook and Twitter (social networking websites); (2) participants’ opinions on the use of virtual communication in patient care; and (3) participants’ views of development of a regulated platform for the use of virtual communication to aid patient care.

### Data Collection and Analysis

All responses to the online questionnaire were automatically recorded through the Lime Survey platform and downloaded to SPSS software version 19.0. A descriptive analysis was obtained followed by a bivariant analysis to detect statistical associations between the participant independent variables and their standpoint (with/against/neutral) on the use of social media in the medical setting. A *P* value of .05 or less was considered statistically significant.

## Results

### Participants

Of the 834 doctors who received the invitation email, 270 participated in the survey but only the 238 physicians who fully completed the survey were included in our data analysis, yielding an overall response rate of 28.5%. The participants were almost equally distributed by gender with males making up 55.0% (131/238) of the responders. The mean age of the participants was 39.4 (SD 13.3) years and the majority of responders were attending physicians (57.1%, 136/238). Most participants were from medical specialties (183/238, 76.9%), which included internal medicine and its various subspecialties: family medicine, radiology, dermatology, psychiatry, pediatrics, pathology, emergency medicine, neurology, and laboratory medicine. The responders’ demographics and characteristics are reported in Table 1.

### Online App Usage Patterns

All physicians reported any-purpose use of online apps in the last 6 months. All participating physicians used email in the given time frame. The second most common form of any-purpose online app for communication was WhatsApp (230/238, 96.6%) followed by Facebook (177/238, 74.4%), and to a lesser extent LinkedIn (91/238, 38.2%) and Twitter (54/238, 22.7%). The use of email and LinkedIn for professional purposes was higher than their use for personal purposes (230/238, 96.6% vs 167/238, 70.2% and 79/238, 33.2% vs 34/238, 14.3%, respectively). Physicians used the remaining online apps more frequently for personal purposes (Table 2).

### Attitudes and Opinions Toward the Use of Online Apps by Physicians

The use of online apps including social media in a professional setting was regarded to aid the communication between different physicians according to 70.2% (167/238) of our participants. Only 42.4% (101/238) of the respondents believed that the use of online apps including social media can be a beneficial tool for patient education. Furthermore, the idea that this virtual form of communication can be used to improve patient health and treatment compliance was only shared by 34.0% (81/238) of our respondents. Approximately half of the participants believed that the use of these online apps and social media can be of use for patients to communicate with one another to share experiences and receive reassurance about their medical condition (Table 3).

**Table 1:** Physician demographics and characteristics (N=238).

Demographics and characteristics		Participants
Age (years), mean (SD)		39.4 (13.3)
**Gender, n (%)**		
	Male	131 (55)
	Female	107 (45)
**Marital status, n (%)**		
	Single	102 (42.9)
	Married	133 (55.9)
	Divorced	2 (0.8)
	Widowed	1 (0.4)
**Specialty, n (%)**		
	Medicine	183 (76.9)
	Surgery	55 (23.1)
**Years in practice, n (%)**		
	<5 years	112 (47.1)
	5-10 years	29 (12.2)
	10-15 years	28 (11.8)
	15-20 years	17 (7.1)
	>20 years	52 (21.8)
**Number of patients seen weekly, n (%)**		
	<10	15 (6.3)
	10-20	52 (21.8)
	20-40	80 (33.6)
	40-60	44 (18.5)
	>60	47 (19.7)
**Medical status, n (%)**		
	Resident/fellow	102 (42.9)
	Attending	136 (57.1)

**Table 2:** Online app use according to purpose.

Online app forms	Any-purpose use, n (%)		Personal purpose, n (%)		Professional purpose, n (%)	
	Yes	No	Yes	No	Yes	No
Email	238 (100)	0 (0)	167 (70.2)	71 (29.8)	230 (96.6)	8 (3.4)
WhatsApp	230 (96.6)	8 (3.4)	222 (93.3)	16 (6.7)	166 (69.7)	72 (30.3)
LinkedIn	91 (38.2)	147 (61.8)	34 (14.3)	204 (85.7)	79 (33.2)	159 (66.8)
Facebook	177 (74.4)	61 (25.6)	172 (72.3)	66 (27.7)	20 (8.4)	218 (91.6)
Twitter	54 (22.7)	184 (77.3)	52 (21.8)	186 (78.2)	19 (8.0)	219 (92.0)

**Table 3:** Physician standpoint on the potential benefits of virtual communication.

Benefits of virtual communication	Standpoint, n (%)	
	Yes	No
Provides quicker and more efficient communication between physicians	167 (70.2)	71 (29.8)
Decreases nonurgent telephone calls	126 (52.9)	112 (47.1)
Reassures patient about disease	120 (50.4)	118 (49.6)
Allows patients to share similar experiences (eg, on blogs and forums)	112 (47.1)	126 (52.9)
Allows better patient education	101 (42.4)	137 (57.6)
Creates continuous access to health care system	87 (36.6)	151 (63.4)
Helps monitor patients’ health and improve treatment compliance	81 (34.0)	157 (66.0)
Allows physicians to handle larger number of patients	40 (16.8)	198 (83.2)

**Table 4:** Physician standpoint on the potential barriers of virtual communication.

Barrier	Standpoint, n (%)	
	Yes	No
Raises medicolegal issues	186 (78.6)	51 (21.4)
Patients are not able to judge authenticity of information provided online	177 (74.4)	61 (25.6)
Provides false patient reassurance	171 (71.8)	67 (28.2)
Invades physician privacy	169 (71.0)	69 (29.0)
Is unprofessional	127 (53.4)	111 (46.6)
Delays patients from visiting health care professionals	124 (52.1)	114 (47.9)
Effects patient-physician confidentiality	115 (48.3)	123 (51.7)
Increases patient anxiety	110 (46.2)	128 (53.8)
Increases physician workload	98 (41.2)	140 (58.8)
Invades patient privacy	94 (39.5)	114 (60.5)

The barriers to the use of virtual communication as a means of communicating with patients according to our participating physicians are reported in Table 4. The majority of the participants (186/238, 78.6%) felt that this mode of communication can result in medicolegal issues, and 71.0% (169/238) felt that it was a breach of privacy. Most physicians also believed that online sources of information for patients are problematic because patients are not sufficiently qualified to judge the authenticity of information presented to them by social media.

The results demonstrating the effect of participant characteristics on their attitudes toward relying on virtual communication in medical settings is shown in Table 5. Physicians’ attitudes were nearly equally distributed between those who were strictly against the use of the online apps and social media (113/238, 47.5%) in their daily profession and those who were with or neutral with such use (125/238, 52.5%). Physicians who were of male gender (*P*=.003), of older age (*P*=.02), faculty members (*P*<.001) and in the surgical specialty (*P*=.03) were more likely to have positive attitudes toward the use of online apps and social media. A prior positive experience with such use in their interactions with their patients was also shown to be a positive predictor of a positive attitude. Years in practice or the number of patients seen per week did not influence physician attitude (*P*=.07 and *P*=.58, respectively).

The most common method adopted by participating physicians in dealing with unwanted online communication with patients was by adjusting their privacy settings on their online apps (143/238, 60.0%), closely followed by ignoring a friend request (105/238, 52.1%). Only 13.4% (32/238) of the participants reported blocking patients as their way to avoid communicating with patients on social media.

When asked about awareness of existing current guidelines on physician-patient communication and only 6.7% (16/238) of the participants answered “yes” and 81.5% (195/238) felt that guidelines are necessary to facilitate and direct this form of communication (Table 6).

**Table 5:** Influence of participant characteristics on standpoint toward using virtual communication.

Variable		Total (N=238)	Standpoint			*P* value
			With (n=63)	Neutral (n=62)	Against (n=113)	
Age (years), mean (SD)		39.36 (13.27)	43.33 (13.13)	38.19 (12.22)	37.78 (13.55)	.02
**Gender, n (%)**						.003
	Male	131 (55.0)	46 (73.0)	28 (45.2)	57 (50.4)	
	Female	107 (45.0)	17 (27.0)	34 (54.8)	56 (49.6)	
**Marital status, n (%)**						.52
	Single	105 (44.1)	24 (38.1)	28 (45.2)	53 (46.9)	
	married	133 (55.9)	39 (61.9)	34 (54.8)	60 (53.1)	
**Specialty, n (%)**						.03
	Medicine	183 (76.9)	41 (65.1)	50 (80.6)	92 (81.4)	
	Surgery	55 (23.1)	22 (34.9)	12 (19.4)	21 (18.6)	
**Years in practice, n (%)**						.07
	<5 years	112 (47.1)	21 (33.3)	31 (50.0)	60 (53.1)	
	5-20 years	74 (31.1)	22 (34.9)	21 (33.9)	31 (27.4)	
	>20 years	52 (21.8)	20 (31.7)	10 (16.1)	22 (19.5)	
**Medical status, n (%)**						<.001
	Resident/fellow	102 (42.9)	14 (22.2)	28 (45.2)	60 (53.1)	
	Attending	136 (57.1)	49 (77.8)	34 (54.8)	53 (46.9)	
**Experience in past, n (%)**						<.001
	With	70 (29.4)	50 (79.4)	12 (19.4)	8 (7.1)	
	Neutral	112 (47.1)	13 (20.6)	43 (69.4)	56 (49.6)	
	Against	56 (23.5)	0 (0)	7 (11.3)	49 (43.4)	

**Table 6:** Methods of avoiding patient communication on online apps and social media.

Method of avoidance	n (%)
Adjust privacy settings	143 (60.1)
Ignore friend requests	124 (52.1)
Ignore emails	105 (44.1)
Block people of social media	32 (13.4)

## Discussion

This survey on the perception of the use of virtual communication in the medical setting at an academic center in Lebanon showed that the majority of participants are not opposed to the idea of using online apps and social media and communication in an interdisciplinary manner to communicate with other physicians. However, most felt that using it as a tool to communicate with patients would not result in an improved physician-patient interaction. The main reasons voiced by physicians for holding this standpoint were that they felt this mode of communication could result in increased medicolegal issues, could cause a breach in privacy, is unprofessional, and could cause a delay in patients visiting health care professionals. Trends in the use of social media for patient communication among physicians at our medical center compare to those of physicians in Australia [9] and the United States [10]. According to a study carried out in Australia, only a minimal number of physicians use social media as part of their professional careers [9]. A national survey in the United States showed that almost half of physicians and medical students did not believe that online services and communications could improve patient-physician communication [10].

Our results also demonstrated that older physicians and practicing/attending physicians where more accepting of the idea of using online apps and social media as a form on patient-physician communication. This is contrary to what we would expect. This could be because older physicians are more confident in their ability to maintain personal boundaries between their patients and themselves even through social media and online communication. This study also showed that physicians in the surgical speciality were less opposed to the use of social media for patient interaction. Surgical physicians may favor this form of communication because it facilitates short-term follow-up postoperatively and provides a more effective form of communicating preoperative preparation instructions [11]. Online postoperative follow-ups are more time efficient and are effective in determining if patients need further personal care [12]. The benefits of online postoperative patient follow-up may not only be beneficial for short-term follow-ups. An ongoing study is being conducted to determine benefits of online long-term follow-up of patients’ post-bariatric surgery in attempt to provide continued support to patients who may otherwise be lost to follow-up [13].

Patients with portable devices can communicate with physicians and clinicians through email, text messages, chat, and Web apps, and receive advice and recommendations without visiting the hospital or clinic resulting in better accessibility to health care and faster access to test results and reports [2]. Additionally, social media and online apps as a means of relaying messages and reminders to patients can improve the field of preventive medicine by increasing vaccination rates [14] and improving smoking cessation rates [15]. Virtual communication between health care providers and patients may also result in improved patients’ understanding and compliance to physician instructions [6]. Not to mention better adherence to medications in patients with chronic diseases by providing a fast track to prescription refills [2]. However, not only physicians resist this shift in health care practice, but also patients. Patients also seem less willing to use online apps and social media and networking for health care services as compared to non-health care services [16].

Although the potential pitfalls to online physician-patient communications are concerning, the American College of Physicians published a policy statement titled “Online Medical Professionalism: Patient and Public Relationships” [17] in 2013. The policy statement emphasizes that online physician-patient communication should supplement rather than replace the traditional face-to-face encounter. To maintain patient confidentiality, protocols for storage and transfer of patient information must be established and secure networks employed. To preserve both personal and patient privacy, physicians are recommended to avoid “friending” patients on social media and to use professional profiles for communication rather than personal profiles. To minimize medicolegal issues that may arise from online communication, it is suggested that online communication only take place between a physician and a patient with an already established face-to-face relationship and prior discussion and agreements to be set. In fact, several professional opinions have advocated against online communication sharing of medical advice with an unknown patient [18]. Because of the novelty of this form of physician-patient communication, physicians do not have a lot of experience in dealing with online ethical dilemmas. Adhering to available guidelines addressing this issue is fundamental. At our academic medical center, only 6.7% (16/238) of the participants were aware of current existing guidelines on online patient-physician interaction.

Limitations of the study include a low survey response rate and survey distribution being limited to AUBMC, which significantly limits the generalizability of the study. Additionally, this study is prone to self-selection bias, in that individuals who are more comfortable using online communication were more likely to access the online questionnaire. Hence, our study could be underestimating physician disapproval toward the use of social media as a means of communicating with patients.

In conclusion, physicians at an academic medical center in Lebanon were reluctant to use online apps and social media as a form of communication with patients. Our study is the first to evaluate the usage and to describe the views of physicians toward the use of virtual communication via various online apps and social media in their daily interaction with patients. Our results provide novel information to the region, which could help in the development of polices and strategies that will result in better online physician-patient communication and make this form of communication less intimidating to physicians. Institutions need to start embracing this new form of physician-patient communication rather than fight it.

## Abbreviations

AUBMC: American University of Beirut Medical Center
